# A framework for secondary cognitive and motor tasks in dual-task gait testing in people with mild cognitive impairment

**DOI:** 10.1186/s12877-018-0894-0

**Published:** 2018-09-03

**Authors:** Susan W. Hunter, Alison Divine, Courtney Frengopoulos, Manuel Montero Odasso

**Affiliations:** 1 School of Physical Therapy, Elborn College, University of Western Ontario, Room 1588, London, ON N6G 1H1 Canada; 2 Schulich School of Medicine & Dentistry, Division of Geriatric Medicine, University of Western Ontario, London, Ontario Canada; 3 Gait & Brain Lab, Lawson Health Research Institute, London, Ontario Canada; 4 Parkwood Institute, Main Building, 550 Wellington Road, Rm A2-129, London, ON N6C 0A7 Canada

**Keywords:** Gait, Cognitive dysfunction, Aged

## Abstract

**Background:**

Cognition is a key factor in the regulation of normal walking and dual-task gait assessment is an accepted method to evaluate the relationship. The objective of this study was to create a framework for task complexity of concurrent motor and cognitive tasks with gait in people with mild cognitive impairment (MCI).

**Methods:**

Community-dwelling people with MCI (*n* = 41, mean age = 76.20 ± 7.65 years) and cognitively normal controls (n = 41, mean age = 72.10 ± 3.80 years) participated in this study. Gait velocity was collected using an instrumented walkway under one single task and six combined tasks of motor and cognitive activities. The cognitive cost was the difference between the single gait task and each of the concurrent motor and cognitive challenges. A repeated two-way measure ANOVA assessed the effect of cognitive group and walking test condition for each gait task test.

**Results:**

Gait velocity was significantly slower in the MCI group under all tasks. For both groups, the concurrent motor task of carrying a glass of water conferred a challenge not different from the cognitive task of counting backwards by ones. Performance of the complex cognitive task of serial seven subtractions reduced gait velocity in both groups, but produced a greater change in the MCI group (31.8%).

**Conclusions:**

Not all concurrent tasks challenge cognition-motor interaction equivalently. This study has created a framework of task difficulty which allows for the translation of dual-task test conditions to future research and clinical practice to ensure the accuracy of assessing patient deficits and risk.

## Background

Falls in older adults are an important public health concern, as at least 35% of adults over the age of 65 years fall each year [1]. An important risk factor for falls that feature prominently in fall prevention guidelines is gait impairment [1]. Gait is a complex task involving the integration of information from multiple systems to maintain postural stability and the research demonstrates that cognition plays a key role in the normal regulation of walking [2]. The inability to maintain stability while responding to commands or attending to additional tasks while walking can lead to postural instability and an elevated fall risk [3–5].

The dual-task paradigm, which consists of the simultaneous performance of another activity while walking is an accepted way to evaluate the interaction between cognition and mobility [6]. Performance will be reduced on either the primary task, the secondary task or both if the cognitive load of performing the simultaneous tasks is greater than cognitive capacity [2]. The dual-task paradigm is relevant as most normal daily activities require the simultaneous performance of motor and cognitive tasks. Therefore, this testing format may identify limitations present during ordinary daily activities that lead to falls [7]. The cognitive cost on gait, or difference between the single and combined tasks, quantifies the demands on cognitive resources and an increased cognitive cost is associated with an increased falls risk [8]. Therefore, performance decrements during dual-task testing are related to the individual’s ability to allocate cognitive resources [2, 7], which is dependent on the nature of the cognitive task and the difficulty of the mobility task [9].

People with MCI are an important patient population for the clinical use of dual-task gait testing as they have subtle objective cognitive impairment, gait deficits that are sub-clinical under usual gait assessment procedures [10] and possess relatively good physical function but have a high risk of falls and injurious falls [11, 12]. The use of dual-task testing has a critical role to play in the areas of MCI diagnosis and the identification of specific gait deficits for initiation of rehabilitation interventions [13]. The Gait & Brain Study, a prospective cohort study investigating gait as a biomarker to identify risk of conversion to dementia among people with MCI, has demonstrated that deficits in dual-task gait testing using secondary cognitive challenges are associated with conversion to dementia [14].

Dual-task gait testing can incorporate several types of tasks to be performed simultaneously with walking, including mental tracking, verbal fluency or another motor task [15]. Indeed, cognitive cost on gait is not constant and will vary with the postural task, so not all secondary tasks will interact with postural control processing in the same way under the dual-task paradigm [7]. Different cognitive tasks in dual-task testing have shown varying effects in people with MCI; specifically, increasing cognitive demands have greater sensitivity for differentiating people with MCI from cognitive healthy older adults [13].

The challenge posed by the literature has been in formalizing the gradation of task complexity. Bahureska et al., [13] demonstrated that when using cognitive tasks with walking, counting backwards by sevens is more complex than verbal fluency, yet counting backwards by ones is no different than single-task walking for differentiating gait effects in people with MCI from cognitively healthy controls. As a result, it cannot be assumed that dual-task test protocols are interchangeable when identifying deficits or that participants are working at or near capacity under a single standard test protocol in order to uncover deficits. There is a lack of recommendations for selecting the most appropriate dual-task protocol, which concurrent motor or cognitive task with gait, to use in clinical practice.

The study by Bahureska et al., [13] provided valuable information on the differential effects of different cognitive tasks in dual-task testing across several gait parameters in people with MCI. A framework of task complexity to progressively increase cognitive demands in a structured way to titrate load in testing would benefit translation of the research into clinical practice [9]. Cognitively under-challenging an individual may result in missing key risk information that may impact initiation of treatment to reduce the risk of adverse outcomes. As such it is necessary to understand and formally outline gradation of task difficulty for a patient population.

Additionally, the effect of concurrent motor tasks and the relative effects of this type of task compared to cognitive secondary tasks with gait among people with MCI has not been evaluated. In a clinical setting the use of either a secondary motor or cognitive challenge can be used by rehabilitation professionals for the evaluation of falls risk [9]. Use of an additional motor task, such as carrying a glass of water, is a means to increase the complexity of the gait task and is not encumbered by literacy levels, language, or speech problems that may impact the use of secondary cognitive tasks.

The primary objective of this study was to evaluate the change in velocity and cognitive cost for different secondary tasks, both cognitive and motor, in people with MCI in order to provide a framework for complexity in dual-task testing. We hypothesized that as an indicator of attentional capacity, dual-task protocols using different secondary tasks will load the attentional system differently and effects will not be uniform across protocols due to novelty and complexity of the task such that cognitive demands relative to the cognitive capacity of the individual will adversely influence how well motor and cognitive processing is performed in people with MCI and 2) the cognitive cost will be greater across all test conditions for older adults with MCI compared to cognitively normal controls.

## Methods

### Study subjects

Study participants were convenience samples from two groups - older adults newly diagnosed with MCI and older adults who were cognitively normal. Participants with MCI were recruited from Aging Brain Memory Clinic at Parkwood Hospital at the University of Western Ontario and the normal controls by newspaper advertisement and from a community fitness program. The participants included in this study were a subsample of data collected from the baseline assessment of a large cohort study, the “Gait & Brain Study” [16] (clinicaltrial.gov #NCT03020381). This subsample of participants were the only group from the bigger cohort to perform all the gait testing outlined below.

Inclusion criteria for the MCI group was a recent diagnosis of MCI, aged 65 years and older and independent ambulators (e.g., no assistant of another person required and do not use a mobility aid). Participants fulfilled the criteria for MCI if they had a subjective memory complaint, a report of cognitive deterioration from the patient and/or family; objective cognitive impairment in one or more cognitive domains; preserved activities of daily living; and absence of clinical dementia [17]. The standard diagnostic protocol for cognitive testing used the Montreal Cognitive Assessment (MoCA) [18], scores less than 26 considered abnormal, and a score of 0.5 on the Clinical Dementia Rating Scale (CDR). Control participants were recruited by newspaper advertisement and from a community fitness program. The inclusion criteria for the control group were: no subjective memory complaints, normal performance on cognitive tests, absence of functional impairment, and ability to walk independently. Exclusion criteria for all the participants were lack of English proficiency, Parkinsonism, or any neurologic disorder with residual motor deficits (e.g., stroke), musculoskeletal disorders (e.g., severe osteoarthritis of lower limbs) or history of knee/hip replacement affecting gait performance at clinical examination, use of psychotropics (e.g., neuroleptics or benzodiazepines), and major depression.

The project was approved by the University of Western Ontario Research Ethics Board for Health Sciences Research Involving Human Subjects.

### Medical and cognitive assessments

Participants in both groups received the same study assessment procedures. Participants provided socio-demographic information (i.e., age and education), medical information (i.e., co-morbidities and medications) and physical functioning (i.e., physical activity level and activities of daily living).

### Quantitative gait assessment

Gait performance was assessed using a portable electronic walkway system (GAITRite® System, CIR Systems Inc. Franklin, NJ.), which was 600 cm in length and 64 cm in width for the automated measurement of spatiotemporal gait parameters. Start and end points were marked on the floor with tape one meter from either end of the mat to avoid the recording of acceleration and deceleration phases. Each participant performed one practice trial of walking.

All gait testing was performed at self-selected usual preferred walking speed. Dual-tasks were evaluated within the categories of mental tracking (serial subtractions by ones (DT-counting1), serial subtractions by sevens (DT-counting7)), verbal fluency (naming types of animals out loud, DT-animals) and a manual motor task (carrying a glass of water on a tray with one hand, DT-motor). Lastly, gait was evaluated under combined cognitive/manual test conditions (multi-tasking or MT) of serial subtractions by ones and serial subtractions by sevens while carrying a glass of water on a tray with one hand (MT-motor&counting1 and MT-motor&counting7 respectively). During the performance of the mental tracking tests and the combined manual/cognitive tests there was no instruction to prioritize gait or the associated cognitive task. Reliability has been previously established for the use of the gait assessment protocol [19]. The order that the single, dual-task and multi-task test were completed in was randomized to control for the effects of learning and fatigue.

For the purpose of the knowledge translation perspective, gait velocity and cognitive cost on gait were the main parameters of interest as these are easily calculated in clinical settings with minimal equipment. In older adults, a valid and reliable sign of fall risk is gait velocity with a threshold of less than 1.0 m/s [20]. Cognitive cost was calculated for each dual-task and multi-task test condition based on the difference in velocity values from the single-task and each dual-task or multi-task condition as a percentage of the single-task value.

### Data analysis

The main analysis involved a comparison of performance between groups using a two-way repeated measures analysis of variance (ANOVA) general linear model on group (MCI vs. control) and gait task for gait velocity and gait cost, adjusted for age. When interactions were non-significant main effects were assessed. If the main effect within groups was significant, a one-way repeated measure ANOVA was performed to evaluate change in velocity across each of the gait test conditions. If the main effect of test condition was statistically significant, post-hoc pair-wise comparisons were performed to evaluate significant differences between groups on test conditions.

The gait cost was calculated as the percentage change in velocity from the single-task of usual walking pace to the dual-task tests (DT-animals, DT-motor, DT-counting1, DT-coutning7) and the simultaneous multi-tasks (MT-motor&counting1, MT-motor&counting7):

$$ Cognitive\ cost\  on\  gait\%=\Big[\left(\left({velocity}_{single- task}-{velocity}_{dual- task\ or\ multi- task\Big)}/{velocity}_{single- task}\right)\times 100\%\right]. $$

The same analyses were performed on the outcome of gait cost. All statistical analyses were performed with SPSS, version 23.0.

## Results

Forty-one older adults with MCI and 41 controls participated in the study. Healthy controls and MCI participants differed on age and as expected on cognitive status (MoCA). (Table 1).

**Table 1:** Participant demographics and characteristics by cognitive status group

Variable	Controls (*n* = 41)	MCI (*n* = 41)	*p*-value*
Age in years (SD)	72.10 (3.80)	76.20 (7.65)	< 0.001
Female, n (%)	33 (80.5%)	23 (56.1%)	0.032
Height (cm)	160.09 (22.48)	166.26 (9.14)	0.099
Body Mass Index (kg/cm^2^)	27.39 (4.01)	25.78 (3.79)	0.065
Years of education (SD)	14.12 (3.59)	12.93 (3.07)	0.123
Number of prescribed medications (SD)	5.56 (3.51)	6.13 (3.47)	0.462
Number of comorbidities (SD)	3.78 (2.30)	4.08 (2.80)	0.598
Falls in previous 12 months, n (%)	8 (19.5%)	9 (22.0%)	0.786
Fear of falling (yes, %)	5 (12.2%)	11 (26.8%)	0.100
MoCA (SD)	28.68 (1.19)	23.10 (2.36)	< 0.001

MCI, mild cognitive impairment; MoCA, Montreal Cognitive Assessment; SD, standard deviation; *, statistically significant at *p* < 0.05

Gait velocity decreased for both groups with the addition of motor and cognitive tasks singly and in combination during multi-task testing. The two-way ANOVA was significant on group, task and the interaction of group x task. (Table 2) Pair-wise comparison between groups demonstrated that gait velocity was significantly different between controls and people with MCI (*p* < 0.001), with controls ambulating at a higher velocity than the MCI group in each task condition.

**Table 2:** Differences in gait velocity and cognitive cost between controls and those with Mild Cognitive Impairment

Gait Test	Velocity (m/s)			Cognitive Cost (%)		
	Controls (n = 41)	MCI (*n* = 41)	t-test *p*-value*	Controls (*n* = 41)	MCI (*n* = 41)	t-test *p*-value
Usual gait	1.23 (0.19)^a^	1.13 (0.20)^a^	0.023			
DT-motor	1.21 (0.17)^a^	1.05 (0.17)^a^	< 0.001	1.2 (10.4)^a^	5.8 (10.6)^a^	0.055
DT-counting1	1.19 (0.19)^a,†^	1.00 (0.24)^a,‡^	< 0.001	2.6 (10.1)^a,‡^	11.7 (12.9)^b,†^	< 0.001
MT-motor&counting1	1.15 (0.17)^b,‡^	0.95 (0.21)^b,‡^	< 0.001	5.9 (13.9)^a,‡^	15.7 (12.9)^c,‡^	0.0012
DT-animals	1.11 (0.20)^b,‡^	0.88 (0.28)^c,‡^	< 0.001	10.0 (12.2)^b,‡^	22.3 (16.1)^d,‡^	< 0.001
DT-counting7	1.02 (0.23)^c,‡^	0.73 (0.26)^d,‡^	< 0.001	17.6 (11.5)^c,‡^	34.4 (21.1)^d,‡^	< 0.001
MT-motor&counting7	0.99 (0.22)^c, ‡^	0.75 (0.24)^d,‡^	< 0.001	19.9 (12.9)^c,‡^	32.9 (17.9)^d,‡^	< 0.001
2 way ANOVA*	Group *p* < 0.001 Task *p* < 0.001 Group x Task *p* = 0.005			Group *p* < 0.001 Task *p* < 0.001 Group x Task *p* < 0.001		

*, Bonferroni correction for multiple comparisons; DT-motor, dual-task gait test while carrying glass of water on tray; DT-counting1, dual-task gait test while counting backwards by 1 s; MT-motor&counting1, multi-task gait test of carrying glass of water on tray and counting backwards by 1 s; DT-animals, dual-task gait test while naming animals; DT-counting7, dual-task gait test while counting backwards by 7 s; MT-motor&counting7, multi-task gait test of carrying glass of water and counting backwards by 7 s. Superscript letters (a,b,c,d) denote results of the Bonferroni pairwise within group comparisons across tasks**.** Conditions within the groups that have the same superscript do not differ from one another. Superscript † indicates that there is a significant change in velocity under dual-task testing compared to usual gait and there was a significant change in cognitive cost compared to the cognitive cost under dual-task gait test while carrying glass of water on tray (DT-motor) at the *p* < .05 level and ‡ at the *p* < .001 level

Cognitive cost was different between groups for all test conditions with the MCI group experiencing higher costs than the controls. The exception to this was the DT-motor task, where there was no difference between groups. (Table 2) Not all tasks conferred the same level of cognitive cost within the control and MCI groups. Cognitive cost ranged from 1.2 to 19.9% for controls, while people with MCI had values ranging from 5.8 to 34.4%.

**Fig. 1:**
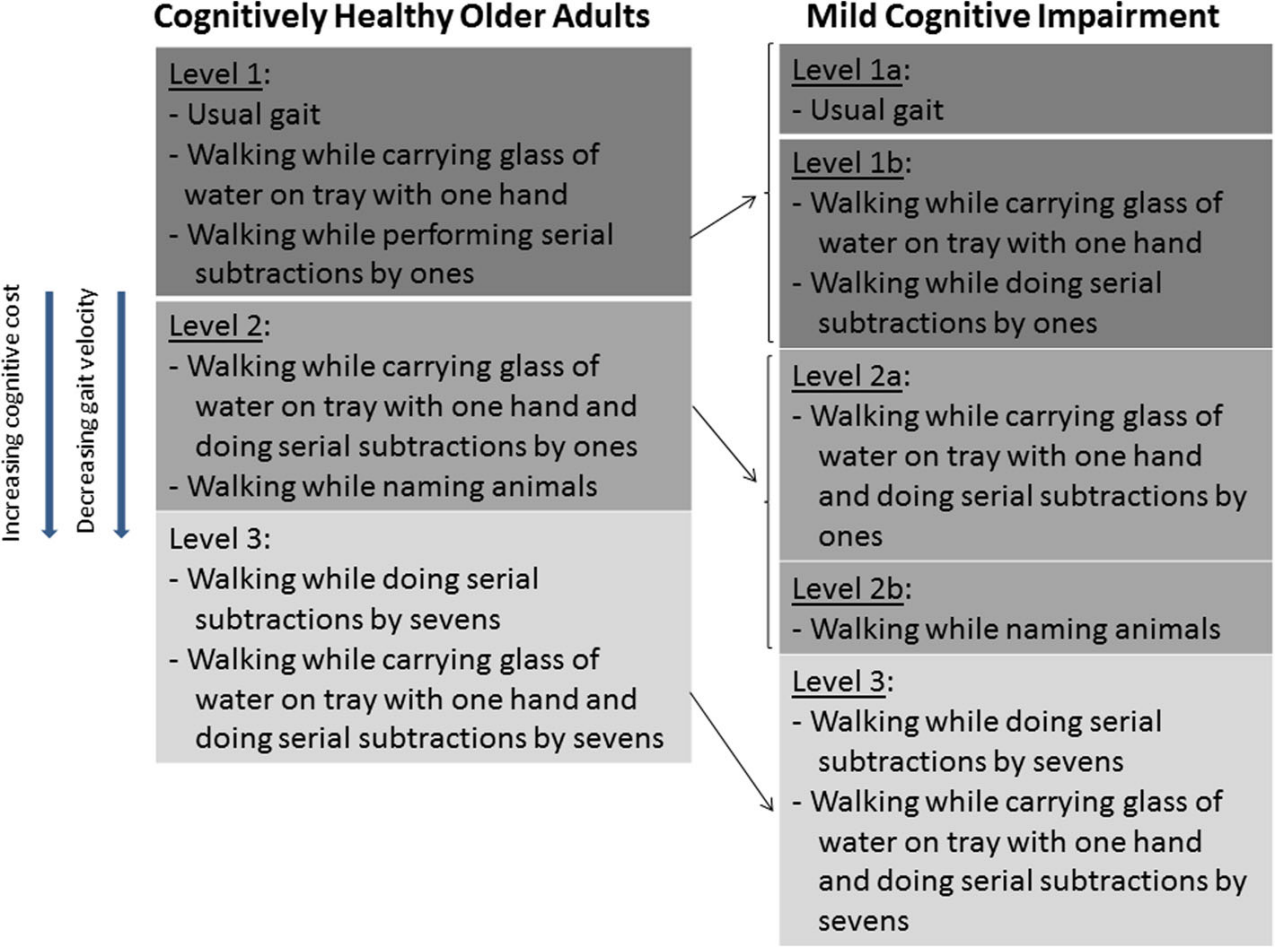
Framework for changes in gait velocity and cognitive cost across gait testing conditions. Note: Gait tests within the same level were not statistically different from one another. Gait velocity decreased on moving from level 1 to level 3. Cognitive cost increased on moving from level 1 to level 3

Post-hoc testing for significant pairwise associations for gait velocity and cognitive cost within the control group and MCI group are presented in Fig. 1. The presentation of the results is meant to denote that gait tests within a level were not statistically significantly different, but gait tests between levels were statistically different (*p* < 0.05). In the control group, there was no difference in gait velocity and cognitive cost for DT-motor, DT-counting1 and usual gait; yet in the MCI group, the DT-motor and DT-counting1 tasks were significantly different to usual gait creating a new level of effect. For the controls, the MT-motor&counting1 and DT-animals were not different, but in the MCI these tests were in separate strata with DT-animals generating a greater cognitive cost and slower velocity. Both groups demonstrated that the effects of the DT-counting7 and MT-motor&counting7 were not different from each other, but exhibited the slowest gait and greatest cognitive cost in each group. Gait velocity dropped below 1.0 m/s for the MCI group at level 2a tasks, while for controls gait velocity was only just below 1.0 m/s at level 3 tasks.

## Discussion

This study has demonstrated the addition of either a motor or cognitive task to walking results in a statistically and clinically relevant loss in gait performance in people with MCI compared to cognitively healthy older adults. Importantly, there is a differential effect on gait velocity and gait cost between concurrent motor and cognitive secondary tasks and among different cognitive tasks in people with MCI. The current findings expand on previous work by Bahureska et al., [13] that identified cognitive tasks that discriminate MCI gait changes from healthy controls, by assessing both cognitive and motor complexity influences on gait performance.

A key finding from our study was an outline for motor and cognitive task difficulty for people with MCI, the type of task complexity framework as proposed by McIsaac et al., [9] that outlines a progressive increase in cognitive demands. These findings build upon previous work by Bahureska et al. [13] through our inclusion of motor tasks to emphasize that tasks are not equivalent with respect to their ability to challenge the interaction between cognition and mobility to identify deficits. Consistent with the findings by Bahureska et al., [13] the dual-task test protocols involving counting backwards by 7 s demonstrated a greater effect than naming animals compared to usual walking for both people with MCI and healthy controls. Our study adds to this ranking by including the effect of a concurrent motor task and multi-tasking (combination of concurrent motor task with the mental tracking dual-task testing protocols).

In dual-task testing the secondary task should be sufficiently challenging that people are working near the limit of their ability. In reporting the results of dual-task testing it is essential to clearly state the nature (i.e., task category as per Al Yahya et al. [15]) and content (e.g., serial subtraction by sevens) of the secondary task performed. It is also important to appreciate that different tasks challenge different cognitive domains, such that tasks involving serial subtractions depend more on working memory and attention, while naming animals is more related to verbal fluency, which relies on semantic memory [6]. Therefore, some people may be proficient with serial subtractions by sevens yet be compromised with a semantic memory task. Gradation of task difficulty can also be performed within a cognitive domain, such that counting backwards by ones is less challenging than counting backwards by sevens. The proposed framework can be used to guide clinicians in choosing tools to progressively increase the cognitive challenge to ensure that patients are working at or close to capacity to uncover deficits. Additionally, the framework can assist clinicians in quantifying change over time. For instance, at time one if a patient can only complete counting backwards by one’s but at time two can now name animals, a clinician is able to track progression of performance.

People with MCI have an increased fall risk and demonstrate greater compromise on physical performance testing compared to cognitively normal older adults [11, 21]. Gait velocity and the change in gait velocity under dual-task testing have been found to be valid methods for evaluating fall risk in older adults without a diagnosis of cognitive impairment [5]. A gait velocity less than 1.0 m/sec is also associated with an increased fall risk among community-dwelling older adults [20]. In our study, even when completing low complexity tasks such as carrying a glass of water or counting backwards by 1 s, gait velocity dropped below 1.0 m/s for those with MCI. Importantly in our study, all of the secondary task combinations produced a statistically significant decrease in gait speed from usual walking for the people with MCI.

Cognitive cost has received less focus than gait velocity in aging research, particularly but not limited for fall risk. Hollman and colleagues [22] reported that a cognitive cost on gait of 20% or higher for gait velocity would have a destabilizing effect and increase the risk for falling. Similarly, a cognitive cost of 20% or higher for gait velocity has been associated with progression to dementia in older adults [14]. Among the participants in our study, it was the MCI group who had cognitive costs clearly in excess of 20%; a finding consistent with previous research demonstrating that people with MCI are at an increased risk for falls [11, 21].

An important strength of this work is that this is the first comparative study the authors are aware of that has evaluated the differential effects of both concurrent motor and cognitive testing, and multitasking with combined simultaneous tasks during gait in the same sample of people with MCI. Our study results also provide an important reference point for the comparison of results between studies that use either manual or cognitive dual-tasks. Lastly, there are some limitations that should be considered. As healthier and higher functioning individuals tend to volunteer for research studies our samples may be biased causing the findings to move towards the null. However, differences between conditions were found suggesting that the results are probable conservative population estimates. Additionally, these results are probably representative of older adults with reasonable education. This study is cross-sectional in nature and therefore determination of which test provides the best predictive validity for the occurrence of future adverse outcomes, such as falls or mobility decline, still needs to be evaluated in a prospective study. The results should not be generalized to other patient populations, as there may be unique disease-related factors that will influence gait performance.

## Conclusions

People with MCI walked at a slower velocity and with a higher cognitive cost in all test conditions compared to cognitively healthy controls. These findings highlight the high vulnerability of gait performance for people with MCI to any extra cognitive challenge. Not all dual-task tests challenge cognition equivalently and the lack of interchangeability, between manual and cognitive secondary tasks and among cognitive tasks, is an important consideration for identification of deficits. This study builds on previous research by including motor and cognitive tasks separately and in combination to provide a framework for graded task difficulty in testing for people with MCI.

## Abbreviations

ANOVA: Analysis of variance
CDR: Clinical Dementia Rating Scale
DT-animals: Dual task test of naming animals
DT-counting1: Dual task test of counting backwards by ones
DT-counting7: Dual task test of counting backwards by sevens
DT-motor: Dual task with motor task of carrying a glass of water on a tray with one hand
MCI: Mild cognitive impairment
MoCA: Montreal Cognitive Assessment
MT-motor&counting1: Multi task test of carrying a glass of water on a tray in one hand and counting backwards by ones
MT-motor&counting7: Multi task test of carrying a glass of water on a tray in one hand and counting backwards by sevens

## References

[1] Summary of the Updated American Geriatrics Society/British Geriatrics Society Clinical Practice Guideline for Prevention of Falls in Older Persons. J Am Geriatr Soc. 2011;59(1):148-57. 10.1111/j.1532-5415.2010.03234.x 21226685

[2] Yogev-Seligmann G, Hausdorff JM, Giladi N (2008). The role of executive function and attention in gait. Mov Disord.

[3] Horak FB (2006). Postural orientation and equilibrium: what do we need to know about neural control of balance to prevent falls?. Age Ageing.

[4] Beauchet O, Annweiler C, Dubost V, Allali G, Kressig RW, Bridenbaugh SA (2009). Stops walking when talking: a predictor of falls in older adults?. Eur J Neurol.

[5] Muir-Hunter SW, Wittwer JE (2015). Dual-task testing to predict falls in community-dwelling older adults: a systematic review. Physiotherapy The Chartered Society of Physiotherapy.

[6] Montero-Odasso M, Verghese J, Beauchet O, Hausdorff JM. Gait and Cognition: A Complementary Approach to Understanding Brain Function and the Risk of Falling. J Am Geriatr Soc. 2012; n/a-n/a 10.1111/j.1532-5415.2012.04209.x PMC3498517 23110433

[7] Woollacott M, Shumway-Cook A (2002). Attention and the control of posture and gait: a review of an emerging area of research. Gait Posture..

[8] Snijders AH, van de Warrenburg BP, Giladi N, Bloem BR (2007). Neurological gait disorders in elderly people: clinical approach and classification.

[9] McIsaac TL, Lamberg EM, Muratori LM (2015). Building a framework for a dual task taxonomy. Biomed Res Int.

[10] Muir SW, Speechley M, Wells J, Borrie M, Gopaul K, Montero-Odasso M (2012). Gait assessment in mild cognitive impairment and Alzheimer’s disease: the effect of dual-task challenges across the cognitive spectrum. Gait Posture.

[11] Liu-Ambrose TY, Ashe MC, Graf P, Beattie BL, Khan KM (2008). Increased risk of failing in older community-dwelling women with mild cognitive impairment. Phys Ther.

[12] Montero-Odasso M, Muir SW, Speechley M (2012). Dual-task complexity affects gait in people with mild cognitive impairment: the interplay between gait variability, dual tasking, and risk of falls. Arch Phys Med Rehabil.

[13] Bahureska L, Najafi B, Saleh A, Sabbagh M, Coon DW, Mohler J (2017). The impact of mild cognitive impairement on gait and balance: a systematic review and meta-analysis of studies using intrsumented assessment. Gerentology.

[14] Montero-Odasso MM, Sarquis-Adamson Y, Speechley M, Borrie MJ, Hachinski VC, Wells J (2017). Association of dual-task gait with incident dementia in mild cognitive impairment: results from the gait and brain study. JAMA Neurol.

[15] Al-Yahya E, Dawes H, Smith L, Dennis A, Howells K, Cockburn J (2011). Cognitive motor interference while walking: a systematic review and meta-analysis. Neurosci Biobehav Rev.

[16] Montero-Odasso M, Oteng-Amoako A, Speechley M, Gopaul K, Beauchet O, Annweiler C (2014). The motor signature of mild cognitive impairment: results from the gait and brain study. Journals Gerontol Ser A Biol Sci Med Sci.

[17] Albert MS, DeKosky ST, Dickson D, Dubois B, Feldman HH, Fox NC (2011). The diagnosis of mild cognitive impairment due to Alzheimer’s disease: recommendations from the National Institute on Aging-Alzheimer’s association workgroups on diagnostic guidelines for Alzheimer’s disease. Alzheimers Dement.

[18] Nasreddine ZS, Phillips NA, Bédirian V, Charbonneau S, Whitehead V, Collin I (2005). The Montreal cognitive assessment. MoCA: a brief screening tool for mild cognitive impairment J Am Geriatr Soc.

[19] Montero-Odasso M, Casas A, Hansen KT, Bilski P, Gutmanis I, Wells JL (2009). Quantitative gait analysis under dual-task in older people with mild cognitive impairment: a reliability study. J Neuroeng Rehabil.

[20] Abellan van Kan G, Rolland Y, Andrieu S, Bauer J, Beauchet O, Bonnefoy M, et al. Gait speed at usual pace as a predictor of adverse outcomes in community-dwelling older people an International Academy on Nutrition and Aging (IANA) Task Force. J Nutr Heal Aging. 2009/11/20. 2009;13:881–889. 10.1007/s12603-009-0246-z PMC12878092 19924348

[21] Delbaere K, Kochan NA, Close JCT, Menant JC, Sturnieks DL, Brodaty H (2012). Mild cognitive impairment as a predictor of falls in community-dwelling older people. Am J Geriatr Psychiatry American Association for Geriatric Psychiatry.

[22] Hollman JH, Kovash FM, Kubik JJ, Linbo RA (2007). Age-related differences in spatiotemporal markers of gait stability during dual task walking. Gait Posture..

